# Supplementary material for: Imported severe malaria and risk factors for intensive care: A single-centre retrospective analysis
Source: PLoS One. 2019 Nov 14;14(11):e0225135. doi: 10.1371/journal.pone.0225135 (PMC6855647; doi:10.1371/journal.pone.0225135)
Supplement: Attachment — Submitted filename: Response to Reviewers.docx [file pone.0225135.s003.docx]

**REVIEWER #1**

This a retrospective single-centre review of imported severe malaria. I have the following comments:
**1) What were the pre-existing admission criteria for admission to IC?**

**A:** The admission criteria adopted for IC admission were:

- Glasgow Coma Scale <11;
- Presence of multiple convulsions;
- Respiratory distress with PaO2/FiO2 Ratio <100);
- Conditions requiring mechanical ventilation and/or non-invasive ventilation;
- Circulatory collapse or shock and/or MAP <70 mmHg, requiring vasopressor drugs;
- Conditions requiring close monitoring and/or prolonged sedation.

We included the IC admission criteria in the manuscript.

**2) A total of 10 cases needing IC are reported although the reason for admission were cerebral malaria (7), shock (6) and ARDS (4) which totals 17, were there any patients that had dual pathologies?**
A: In our study, 7 patients presented more than one clinical picture at the moment of the admission in the IC, therefore the number of the events (17) is greater than the number of patient (10). A sentence was added in the text, accordingly.

**3) ICU vs non-ICU patients had slower parasitemia but did the ICU patients have higher parasitemia to start off with which could explain the slower clearance?**
**A:** As showed in Table 1, there is no difference in basal parasitemia between ICU vs non-ICU (p= 0.811). Moreover, the data is confirmed at the univariate analysis.

**4) 6 patients were reported as requiring vasopressor drugs for shock but there is no mention of fluid resuscitation used. This may relevant particuarly in light of the FEAST study.
A:** According to the protocol of our Institute, the use of vasopressor drugs is secondary to the fluid resuscitation failure.

**5) There were no deaths or sequelae reported yet the conclusions state 'a multidisciplinary approach might be encouraged... in order to improve outcome', what outcome are they trying to improve? Furthermore what and whom should be part of a multi-disciplinary approach'?
A:** As suggested by the reviewer, we modified the conclusion of the manuscript.

**REVIEWER #2**

**This is a simple, concise cross-sectional study of medical record data from patients with severe malaria to assess predictive risk factors associated to the need of intensive care (IC) at a referral hospital in Italy. Authors used five prognostic malaria scores to analyse the need of IC and applyed malaria-specific and general scores to identify severe malaria patients. The manuscript is well written, clear and intelligible. However, authors should make some changes to the manuscript in order to better interpret and conclude the results.**

**1) Authors are recommended to use the STROBE checklist to improve the reporting quality of results.**

**A:** According to reviewer’s suggestion we modified the methods section using the STROBE checklist, which will be uploaded as a supplementary file.

**2) Some limitations of the study should be discussed, such as the low number of patients requiring intensive care, especially if the species was P. vivax. In addition, the cross-sectional design of the study precludes a more conclusive inference from the study results.**
**A:** We thank the reviewer for the suggestions. The text has been modified accordingly, introducing sentences showing some limitations of the study.

**3) The conclusion of the study should be presented more clearly and objectively. In the Summary, for example, the conclusion presented does not translate the results found in the study.**

**A:** The conclusion has been modified accordingly, introducing sentences that reflect the results showed in the text.
