# Supplementary material for: Imported severe malaria and risk factors for intensive care: A single-centre retrospective analysis
Source: PLoS One. 2019 Nov 14;14(11):e0225135. doi: 10.1371/journal.pone.0225135 (PMC6855647; doi:10.1371/journal.pone.0225135)
Supplement: Attachment — Submitted filename: Response to Reviewers.doc.docx [file pone.0225135.s005.docx]

**REVIEWER #2**

1. **Although the authors stated that the study data is entirely available within the manuscript and in supplementary files, I have not had access to it completely.**

**A:** In the previous revision, we have already uploaded the complete database as Supplementary file (page 23, download link). We will upload again the entire database as a supplementary file. If this is not enough, please let us know what we can do further.

**REVIEWER #3:**

This is a cross sectional and retrospective study where clinical records for patients who were admitted with severe malaria from 2007 to 2015 were reviewed and analyzed by the authors. In this revised version of the manuscript, the authors have satisfactorily addressed comments that were raised by two reviewers. The manuscript is technically sound with the data used supporting the conclusion of the paper. All statistical analyses have been done appropriately and rigorously. Nevertheless I have the following concerns with the revised manuscript which the authors need to also look into:

1. **The presentation of the manuscript is intelligible and uses Standard English though it will benefit from some language usage proofreading.**

**A:** As suggested by the reviewer, the manuscript was widely revised for language editing.

2. **The authors need to clearly distinguish between actual recruitment of the patients and use of records for this research study. An example is where they write that they obtained written informed consent from the patients or their next of kin before enrollment into the study without detailing how they did it considering that by the time of this research the patients were not in hospital.**

**A:** As suggested by the reviewer, we modified the text. According to the protocol in use in our Institute, each patient signs an informed written consent at the time of admission for research purposes and epidemiological investigations on infectious and tropical diseases.

3. **Further the authors write that they obtained written informed consent from patients’ next of kin, since all patients are adults aged above 18 years why was consent obtained from next of kin?**

**A:** As suggested by the reviewer we modified the text. In case of person unable (e.g. coma state) to give consent, a relative/next of kin was asked to provide it.

4. **Authors should spell out all abbreviations the first time they are used in the write up – examples are INMI, h, P. vivax etc.**

**A:** As suggested by the reviewer, we modified the text.
